# Molecular characterization of antimalarial resistance and *hrp2/3* deletions in *Plasmodium falciparum* strains from Mtwara, Tanzania (2021–2024)

**DOI:** 10.1186/s12936-026-06028-2

**Published:** 2026-07-28

**Authors:** Piyathida Thapradit, Watcharee Pagornrat, Wanassanan Madmanee, Raweewan Sangsri, Kiattawee Choowongkomon, Salum Mshamu, Deus S. Ishengoma, Arjen M. Dondorp, Lorenz Von Seidlein, Mallika Imwong

**Affiliations:** 1 Department of Molecular Tropical Medicine and Genetics, Faculty of Tropical Medicine, Mahidol University, 420/6 Ratchawithi Rd., Ratchathewi, Bangkok, 10400 Thailand; 2 Department of Biochemistry, Faculty of Science, Kasetsart University, 50 Ngam Wong Wan Rd., Lat Yao, Chatuchak, Bangkok, 10900 Thailand; 3 CSK Research Solutions, Mtwara, Tanzania; 4 Nuffield Department of Clinical Medicine, University of Oxford, Oxford, UK; 5 National Institute for Medical Research, 11101 Dar es Salaam, Tanzania; 6 Department of Biochemistry, Kampala International University in Tanzania, Dar es Salaam, Tanzania; 7 Centre for Tropical Medicine and Global Health, Churchill Hospital, University of Oxford, Oxford, UK; 8 Mahidol Oxford Tropical Medicine Research Unit, Faculty of Tropical Medicine, Mahidol University, 420/6 Ratchawithi Rd., Ratchathewi, Bangkok, 10400 Thailand

**Keywords:** Malaria, *Plasmodium falciparum*, *pfkelch13*, *Pfdhfr*, *Pfdhps*, Histidine-rich protein 2, Malaria rapid diagnostic test, Tanzania

## Abstract

**Background:**

Malaria remains a significant public health challenge in sub-Saharan Africa and Southeast Asia, with *Plasmodium falciparum* responsible for the majority of infections and deaths, particularly among children aged < 5 years. In Tanzania, the widespread use of antimalarial drugs, such as sulfadoxine-pyrimethamine for preventive therapy and artemisinin-based combination therapies as first-line treatment, has prompted concerns about the emergence and spread of drug resistance. The prolonged use of these drugs may have influenced resistance patterns, varying over time. Moreover, some *P. falciparum* samples have been reported as rapid diagnostic test (RDT)-negative despite microscopic confirmation of *P. falciparum* infection, raising questions about whether such false-negative results are due to poor-quality RDT kits or genetic deletions in the parasite.

**Methods:**

This study investigated drug resistance dynamics and potential false-negative RDT results in *P. falciparum* by analyzing molecular markers in *pfdhps, pfdhfr*, *pfkelch13,* and *pfhrp2*/*3*, with the 18S rRNA gene used as a housekeeping control to verify DNA quality and confirm true parasite positivity.

**Results:**

Molecular analysis revealed a decline in the triple *pfdhfr* mutation paired with double *pfdhps* mutations, alongside an increase in mixed *dhfr–dhps* haplotypes (*χ*^2^ = 28.94, *p* < 0.001), indicating ongoing genetic diversification under drug pressure. Wild-type parasites were predominant (56–76%), and most *pfkelch13* mutations were located in the non-propeller region, with no validated or candidate mutations associated with artemisinin-resistance detected. *P. falciparum*-infected samples that were RDT-negative showed a high frequency of both complete and partial deletions in *pfhrp2* and *pfhrp3*, potentially explaining RDT inconsistencies and false-negative results.

**Conclusions:**

*P. falciparum* remains the dominant species in this high-transmission region, showing a persistently high rate of antifolate resistance. Significant associations between triple *pfdhfr* and double *pfdhps* resistance markers were observed between 2021 and 2024. The continued susceptibility to artemisinin emphasizes the need for ongoing molecular surveillance and monitoring of *hrp2/hrp3* deletions to support malaria control strategies in Tanzania.

**Supplementary Information:**

The online version contains supplementary material available at https://doi.org/10.1186/s12936-026-06028-2.

## Background

In Tanzania, 93% of the population resides in areas of malaria transmission. Due to the high transmission rate, over half of the population suffers from repeated malaria infections each year. Malaria is caused by parasites belonging to the genus *Plasmodium*, of which the most prevalent and virulent species is *Plasmodium falciparum*, responsible for 96% of cases. In comparison, the remaining 4% are caused by *P. malariae* and *P. ovale* [1].

*P. falciparum* is showing increasing resistance to crucial antimalarial drugs. At the end of the twentieth century, a combination treatment of sulfadoxine and pyrimethamine (SP) was introduced to replace chloroquine, particularly in young children. SP remains the preventive drug of choice for intermittent preventive therapy (IPT) in both pregnancy (IPTp) and infancy (IPTi), as well as seasonal malaria chemoprophylaxis (SMC), but resistance has been reported in Tanzania since 1996 [2, 3]. Molecular surveillance of SP resistance is based on polymorphisms in the *P. falciparum* genes encoding the enzymes dihydrofolate reductase (*pfdhfr*) and dihydropteroate synthase (*pfdhps*). In Africa, a significant predictor of SP treatment failure is the presence of three *pfdhfr* mutations (N51I, C59R, and S108N) along with two *pfdhps* mutations (A437G and K540E), which confer SP resistance [4–6]. Thus, there is an urgent need for alternative treatments, early detection of resistance, and enhanced monitoring of drug-resistant infections. Artemisinin combination therapy is the first-line treatment for uncomplicated cases of *P. falciparum*. However, since the beginning of the twenty-first century, artemisinin-resistant *P. falciparum* has been reported in the Greater Mekong Subregion, resulting in delayed parasite clearance [7, 8]. Such strains are characterized by a polymorphism in a gene in the propeller region (*pfkelch13*), which serves as a molecular marker of artemisinin resistance in the early ring stage [7, 9–11]. Partial resistance to artemisinin in African countries, such as R561H in Rwanda and C469Y and A675V in Uganda, is an emerging public health emergency [9, 12, 13]. The detection of R561H mutations in *P. falciparum* in the Kagera border region suggests the spread of *pfkelch13* mutations into Tanzania [14].

The early diagnosis of malaria in Tanzania relies on a rapid diagnostic test (RDT) that uses the *P. falciparum* histidine-rich protein 2 (HRP2) as the antigenic marker. These RDTs are highly sensitive, capable of detecting as few as 50 parasites/μL in *hrp2/3* wild-type *P. falciparum* strains [15]. The paralogous protein *pfhrp3* contains tandem repeat regions that share more than 75% sequence homology with *pfhrp2*, and these repeats can be recognized by monoclonal antibodies developed against *pfhrp2*. The presence of *P. falciparum* parasites with deletions in *hrp2/3* has resulted in the reporting of false-negative results in South America and sub-Saharan African countries, including Rwanda, Congo, Kenya, and Eritrea, with Eritrea reporting the highest frequency of *pfhrp2* and *pfhrp3* deletions (80.8% and 92.3%, respectively) [16–21].

This study aimed to determine the prevalence of molecular markers of resistance in *pfkelch13*, *pfdhfr*, and *pfdhps* and frequencies of *pfhrp2/3* deletions using samples, particularly among children aged collected from the Star Home Project in Mtwara, Tanzania [22], between 2021 and 2024.

## Methods

### Study site and dried blood spot (DBS) samples

All samples were obtained from individuals aged under 16 years, including both male and female participants in the Star Home Project. The Star Homes study protocol and ancillary research was approved by the Faculty of Tropical Medicine, Mahidol University (TMEC 24-084), the National Research Ethics Committee of Tanzania (reference NIMR/HQ/R.8a/Vol.IX/3695; June 17, 2021), and the Oxford Tropical Research Ethics Committee (reference #533–20; July 2, 2020). The trial was registered at ClinicalTrials.gov on August 27, 2020 (NCT04529434).

**Fig. 1:**
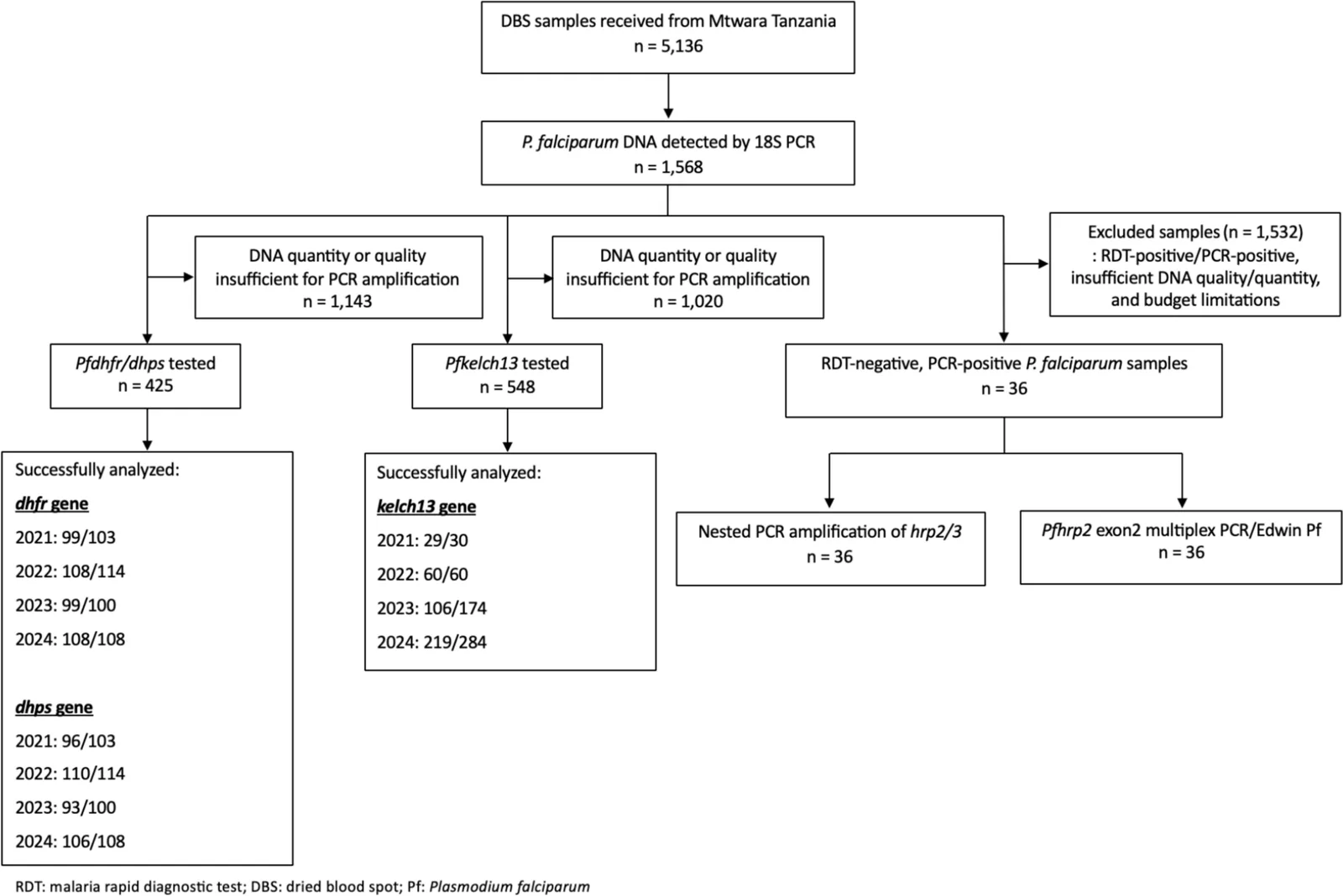
Study workflow and number of analyzed samples

DBS samples were collected across 59 villages in Mtwara District, Tanzania, between 2021 and 2024. Those samples received from the study sites were first screened using a rapid diagnostic test (RDT) for malaria. DNA was extracted from the DBS samples using a QIAamp DNA Mini Kit (QIAGEN, Germany) according to the manufacturer’s instruction.  The extracted DNA was subsequently analyzed by quantitative PCR (qPCR) for molecular detection of Plasmodium falciparum. The qPCR results were then compared with the corresponding RDT results to identify concordant and discordant samples. Samples that were RDT-negative but P. falciparum positive (n = 36) were subsequently selected for pfhrp2/pfhrp3 deletion analysis. The extracted DNA was stored at 4 °C until further analysis (Fig. 1).

### Assessment of *P. falciparum* resistance genes

Parasite densities were estimated based on cycle threshold (Ct) values obtained from a quantitative real-time PCR assay targeting the *Plasmodium* 18S rRNA gene, as previously described by Kamau et al. [23]. Samples with Ct values ≤ 33 were considered to contain sufficient parasite DNA and were selected for amplification by nested polymerase chain reaction (PCR) covering the full length of *pfkelch13* to determine the presence of point mutations. Thermocycling conditions were 94 °C for 5 min; 25 cycles of 94 °C for 1 min, 58 °C for 2 min, and 72 °C for 2 min; followed by a final extension at 72 °C for 10 min [27, 43]. Single nucleotide polymorphisms (SNPs) in *pfdhfr*, *pfdhps*, and *pfkelch13* were detected by Sanger sequencing.

All PCR products were analyzed by agarose gel electrophoresis to confirm successful amplification. PCR products showing clear bands were purified using a FavorPrep Gel/PCR Purification Kit (Favorgen Biotech Corp., Taiwan) according to the manufacturer’s instructions. Purified products were then subjected to Sanger sequencing using an ABI Sequencer (Applied Biosystems, MA, USA) by Macrogen Inc. (South Korea) for detection of mutations at codons 16, 51, 59, 108, and 164 of *pfdhfr* and codons 431, 436, 437, 540, 581, and 613 of *pfdhps*, as previously described [24–26]. The sequencing data were aligned to the *pfdhfr* (chromosome 4; PF3D7_0417200) and *pfdhps* (chromosome 8; PF3D7_0810800) genes of the *P. falciparum* 3D7 reference strain. Sequence editing and analysis were conducted using BioEdit v7.2 (Tom Hall).

Mutations in *pfkelch13* were assessed by nested PCR amplification of a 2283 bp fragment covering the target exon. To ensure accurate Sanger sequencing, the amplified product was subsequently divided into two overlapping fragments (Fragment 1–2 and Fragment 3) prior to sequencing. PCR products were purified using a FavorPrep Gel/PCR Purification Kit (Favorgen Biotech Corp., Taiwan) according to the manufacturer’s instructions and sequenced on an ABI Genetic Analyzer (Applied Biosystems, MA, USA). We aligned the sequencing results against *pfkelch13* of reference strain 3D7 (putative 9PF13_0238 NCBI Reference Sequence (3D7): XM_001350122.1). The PCR conditions and primer sequences for the three genes were as previously described (Ashley et al., 2014) [27] and are provided in Supplementary Table S1 (Additional File 1). Mixed genotypes were defined based on Sanger sequencing chromatograms. A sample was considered mixed when a secondary peak at the same nucleotide position had a height ≥33% of the primary peak. Peaks below this threshold were considered background noise. Genotypes were assigned accordingly as wild-type, mutant, or mixed.

### Pfhrp2 and pfhrp3 genotyping

Samples that tested negative by RDT but were subsequently confirmed positive for *Plasmodium falciparum* by 18S rRNA real-time PCR were selected for further analysis of *pfhrp2* and *pfhrp3* gene deletions using nested PCR with target-specific primers and PCR conditions as previously described Baker et al. [28]. The method described by Gamboa et al. [29] was used to analyze the flanking regions of *pfhrp2* and *pfhrp3*. Primers amplifying loci immediately upstream and downstream of *pfhrp2* (MAL7P1.230 and MAL7P1.228) and *pfhrp3* (MAL13P1.485 and MAL13P1.475) were used to confirm the presence or absence of each gene. In addition, primers targeting exon 2 of both genes were used to further assess gene deletions. Laboratory strains *P. falciparum* 3D7 served as positive control, and Dd2 and HB3 served as negative controls for *pfhrp2* and *pfhrp3* deletions, respectively. To exclude contamination, a PCR assay without template served as the negative PCR control. The PCR cycling conditions for *pfhrp2* were as follows: an initial denaturation at 94 °C for 5 min, followed by 30 cycles of 94 °C for 1 min, 55 °C for 1 min, and 72 °C for 1 min, with a final extension at 72 °C for 10 min. Nested PCR was performed for *pfhrp3* using same conditions; however, the amplification consisted of 30 cycles for the first round and 35 cycles for the second round of PCR.

### Nested PCR for *pfhrp2* exon 2 deletion

To determine the presence of *pfhrp2* exon 2 deletions, 36 *P. falciparum* isolates were analyzed using a multiplex PCR assay targeting *pfhrp2* exon 2 together with the *Plasmodium falciparum* 18S rRNA gene as an internal control for parasite DNA. The analysis primarily focused on RDT-negative samples to investigate potential gene deletions as a cause of false-negative RDT results. In addition, RDT-positive samples (n = 30) were included for comparative analysis and were selected based on sample availability and a range of parasite densities. This approach allowed confirmation of parasite DNA presence while assessing the deletion status of the *pfhrp2* gene and comparing the results with RDT performance in relation to parasite density. Each PCR reaction was prepared in a final volume of 25 µL containing 1 × PCR buffer, 3 mM MgCl_₂_, 125 µM dNTPs, 250 nM of each primer (Additional File 1: Table S1), and 0.4 U Platinum *Taq* DNA Polymerase (Invitrogen, Thermo Fisher Scientific, MA, USA). The PCR cycling conditions were as follows: initial denaturation at 94 °C for 5 min; 30 cycles of denaturation at 94 °C for 1 min, annealing at 60 °C for 2 min, and extension at 72 °C for 2 min; and final extension at 72 °C for 10 min. Laboratory strain *P. falciparum* 3D7 served as the positive control and Dd2 as the negative control for *pfhrp2* deletions. To rule out contamination, a no-template control (NTC) was included in the PCR assay.

### Multiplex qPCR assay for *pfhrp2* and *pfhrp3* deletion confirmation

In accordance with WHO recommendations for the confirmation of *pfhrp2/3* gene deletions, a previously described method by Grignard et al. [30] was conducted to support the detection of gene deletions in *P. falciparum* samples with *pfldh* and *HumTuBB* used as the internal control.

### Protein homology modeling and stability prediction

Three-dimensional protein homology modeling was performed using the AlphaFold Server (https://alphafoldserver.com/), with amino acid sequences as input files. PyMOL v2.5.2 was used to visualize the structure and mutation residues. The protein stability of the Kelch13 propeller domain of each mutant was predicted using the default setting in DUET (https://biosig.lab.uq.edu.au/duet/stability), and the I-Mutant 2.0 web server (https://folding.biofold.org/i-mutant/i-mutant2.0.html) was used to observe changes in stability due to the mutations (ΔΔG; decreased stability: ΔΔG < 0, increased stability: ΔΔG > 0) [31, 32]. The prediction was based on two available crystal structures in the RCSB database (Protein Data Bank ID: 4ZGC and 4YY8), which differed in disulfide bonding and protein sequence length.

### Sequence polymorphism and data analysis

The frequencies of each point mutation in the *pfdhfr*, *pfdhps*, and *pfkelch13* resistance markers, as well as the combined *pfdhfr/pfdhps* haplotypes, were estimated as percentages (number of mutant alleles divided by the total number of successfully genotyped samples) for each year. The χ^2^ test was used to evaluate differences in the frequency of each antifolate resistance marker and compare the distribution of the mutant haplotype across the years. *P*-values ≤ 0.05 were considered statistically significant.

## Results

### Prevalence of *pfdhfr* mutations

**Fig. 2:**
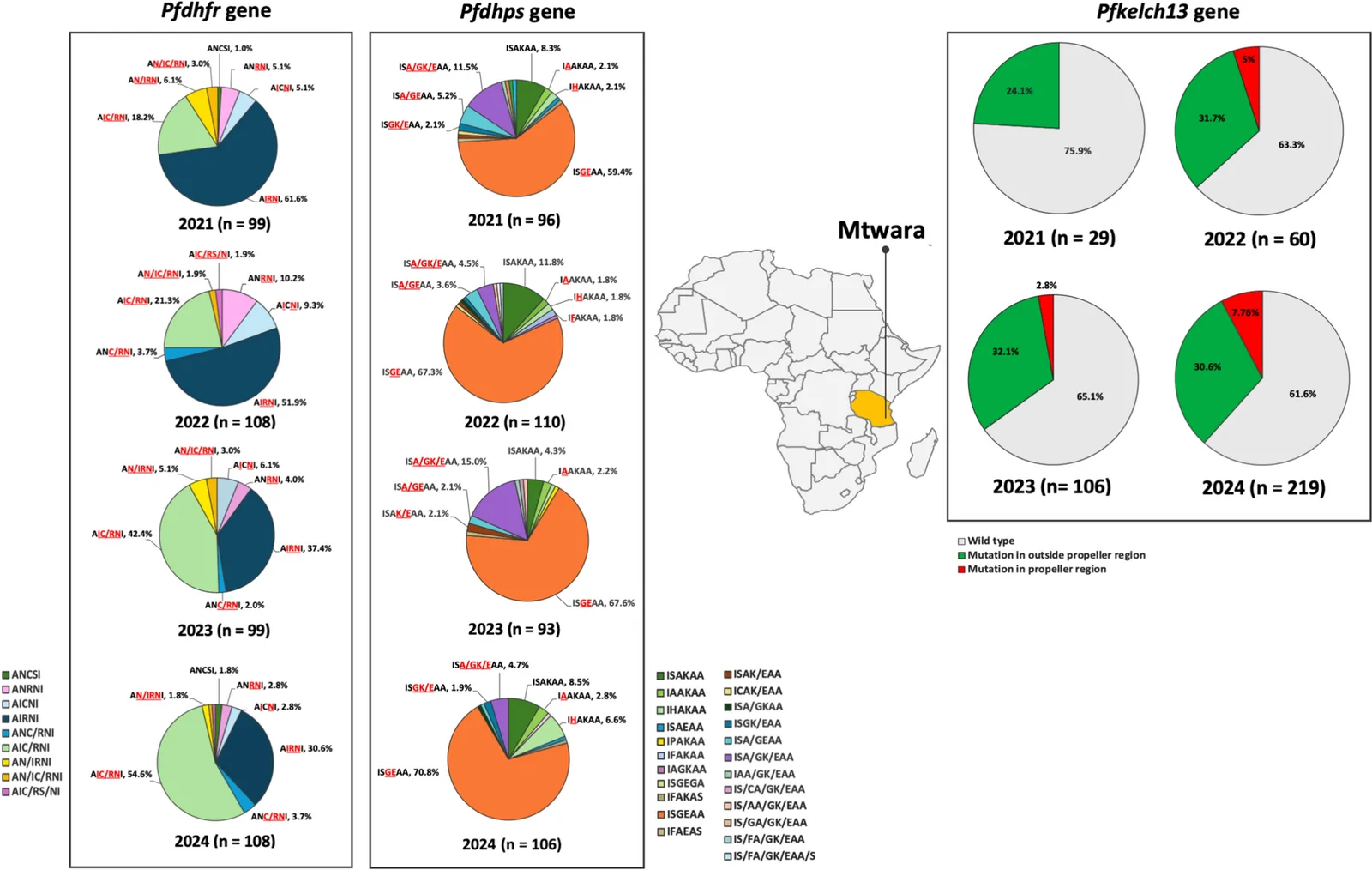
Prevalence of haplotypes of *pfdhfr* and *pfdhps*, and the presence of mutations in *pfkelch13* in the isolates collected from Tanzania (2021–2024)

The most common *pfdhfr* genotype observed in samples collected between 2021 and 2024 was the triple mutant (N51I, C59R, and S108N). The prevalence of this genotype was 61.6% (61/99), 51.9% (56/108), 37.4% (37/99), and 30.6% (33/108) in each study year, respectively. Over the same period (2021–2024), the genotype N51I, C59C/R, and S108N was present in 18.2% (18/99), 21.3% (23/108), 42.4% (42/99), and 54.6% (59/108) samples, respectively. In addition, the prevalence of the genotype N51I, C59C, and S108N was 5.1% (5/99), 9.3% (10/108), 6.1% (6/99), and 2.8% (3/108), respectively. Overall, the prevalence of resistant *P. falciparum* genotypes decreased, while mixed genotypes increased (Fig. 2; See Additional File 1: Table S2).

### Prevalence of *pfdhps* mutations

Double-mutant haplotypes remained the dominant *pfdhps* genotypes, whereas the wild-type allele was rarely detected. Mutations were assessed at codons 431, 436, 437, 540, 581, and 613 (I431V, S436F/A, A437G, K540E, A581G, and A613S/T). In 2021, the double-mutant haplotype ISGEAA was the most prevalent, observed in 59.4% (57/96) of samples, followed by mixed genotypes (ISGK/EAA) in 11.5% (11/96). In 2022, wild-type haplotypes accounted for 11.8% (13/110), while the ISGEAA haplotype predominated, occurring in 67.3% (74/110) of samples. In 2023 and 2024, the ISGEAA haplotype remained dominant, occurring in 67.7% (63/93) and 70.8% (75/106) of samples, respectively (Fig. 2). These findings indicate the prevalence of *pfdhps* double-mutation haplotypes differed significantly throughout the study period (χ^2^ = 48.41,* p* = 0.029; See Additional File 1: Table S3).

### Combination of resistant *dhfr–dhps* haplotypes

Among combined *pfdhfr* and *pfdhps* haplotypes, the triple *dhfr* mutation AIRNI paired with the double *dhps* mutation ISGEAA was the most prevalent. Varied patterns of mutant haplotypes were commonly observed, whereas wild-type parasites were rare (Table 1; See Additional File 1: Supplementary Table S4).

**Table 1:** Prevalence of *dhfr* and *dhps* haplotype combinations in *P. falciparum* isolates from Tanzania (2021–2024)

Year	Category	Haplotype	*n* (%)
2021	Quintuple mutant	AIRNI + ISGEAA	33 (35.1)
(n = 94)		AIC/RNI + ISGEAA	11 (11.7)
2022	Quintuple mutant	AIRNI + ISGEAA	39 (37.5)
(n = 104)		AIC/RNI + ISGEAA	13 (12.5)
2023	Quintuple mutant	AIRNI + ISGEAA	22 (23.9)
(n = 92)		AIC/RNI + ISGEAA	26 (28.3)
2024	Quintuple mutant	AIRNI + ISGEAA	22 (20.8)
(n = 106)		AIC/RNI + ISGEAA	43 (40.6)

The prevalence of the quintuple mutant haplotype (AIRNI + ISGEAA) increased slightly from 35.1% (33/94) in 2021 to 37.5% (39/104) in 2022. Quintuple mixed wild-type haplotypes (AIC/RNI + ISGEAA) were less frequent, observed in 11.7% (11/94) in 2021 and 12.5% (13/104) in 2022. In 2023, the prevalence of mixed wild-type haplotypes (AIC/RNI + ISGEAA) rose to 28.3% (26/92), while AIRNI + ISGEAA declined to 23.9% (22/92). This trend continued in 2024, with mixed wild-type haplotypes increasing further to 40.6% (43/106) and AIRNI + ISGEAA decreasing to 20.8% (22/106). Overall, mixed wild-type haplotypes increased, and classical quintuple mutations decreased over the 4-year study period (χ^2^ = 28.94,* p* < 0.001). These findings may suggest selection of drug-resistant haplotypes under antimalarial drug pressure, with a predominance of mutant genotypes and wild-type haplotypes being rarely detected throughout the study period.

### Prevalence of *pfkelch13* mutations

**Fig. 3:**
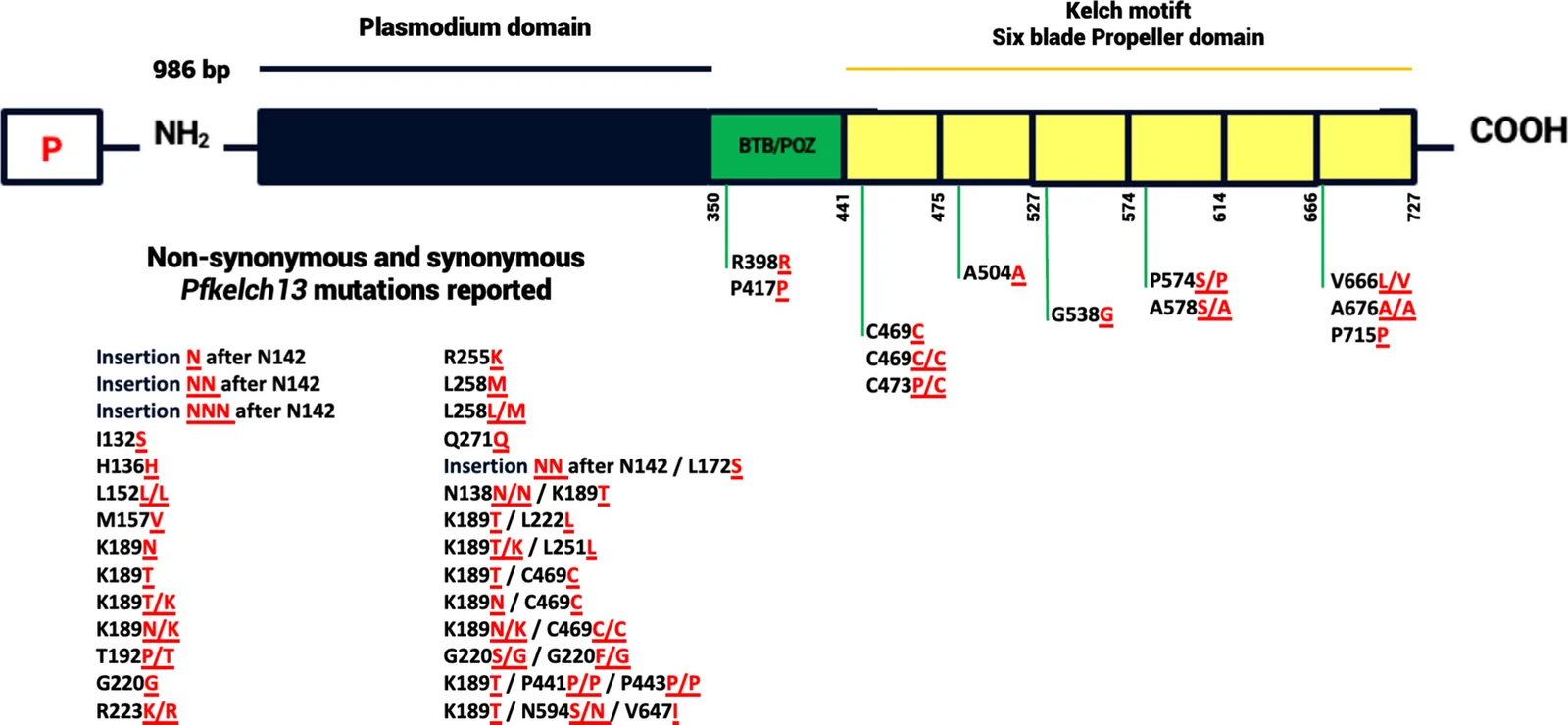
Schematic structure of *pfkelch13* showing the mutations present in the isolates collected from Tanzania

Throughout the study period (2021–2024), the majority of *P. falciparum* isolates were *pfkelch13* wild type, with a prevalence of 75.9% (22/29) in 2021. Mutations were observed in the non-propeller region (Fig. 2), including K189T (20.7%, 6/29) and an asparagine insertion at codon 142 (3.4%, 1/29) in 2021. No mutations in the propeller region associated with artemisinin resistance were detected in 2021. In 2022, 63.3% (38/60) of isolates were wild type. K189T was detected in 15% (9/60) isolates, and K189N and K189T/K in 1 isolate each (1.7%). Other non-propeller polymorphisms included I132S, T192P/T, and R255K in 1 isolate each (1.7%). Asparagine insertions at codon 142 (N, NN, and NNN) were observed in 8.3% (5/60) isolates. Synonymous mutations in the propeller region (R398R, P417P, and C469C) were each detected in 1 isolate (1.7%, 1/60). In 2023, 56.1% (69/106) of isolates were wild type. The most common non-propeller mutation was mixed K189T/K (12.3%, 13/106), followed by K189T (7.6%, 8/106). Propeller region mutations included the synonymous mutation C469C. Among the 219 isolates from 2024, 61.1% (135/219) were wild type. Non-propeller mutations included K189T (13.2%, 29/219), K189T/K (7.3%, 16/219), asparagine insertions at codon 142 (1.8%, 4/219), R255K (1.4%, 3/219), and other combinations. The frequencies of mixed, double, and triple mutations in the propeller region were low (See Supplementary Table 5, in Additional File). No validated or candidate mutations associated with delayed parasite clearance were detected over the 4-year study period. Overall, most mutations occurred in the non-propeller region, with a low prevalence of mutations in the propeller region (Fig. 3; See Additional File 1: Table S5).

### Kelch 13 (K13) protein modeling and stability prediction

The six single mutations (K189N, K189T, C473P, P574S, A578S, and V666L) and one triple mutation (K189T + N594S + V647I) did not alter the K13 protein structure. The K189 residua was located on a large loop of the generated structure, and further stability predictions showed six mutations situated at different locations within a propeller domain (See Additional File 1: Fig. S1). The AlphaFold web server can predict the effect of a single point mutation on protein structure. The triple mutation *kelch13* haplotype had to be excluded from the prediction. As a result, four single mutations reduced the protein stability, as shown by consistently negative ΔΔG values from DUET and I-Mutant 2.0 predictions (Table 2). In vitro biochemical testing is necessary to address the impact of mutations and protein stability.

**Table 2:** Protein stability prediction of the K13 propeller domain by DUET and I-Mutant 2.0

No	Mutation position	DUET^a^ (ΔΔG, kcal/mol)			I-Mutant 2.0^b^ (ΔΔG, kcal/mol)		Stability prediction
		mCSM	SDM	DUET	PDB ID: 4ZGC	PDB ID: 4YY8	
1	C473P	− 1.51	− 1.15	− 1.54	− 1.57	− 1.26	Destabilizing
2	P574S	− 1.70	− 0.55	− 1.51	− 0.90	− 0.90	Destabilizing
3	A578S	− 1.35	− 2.47	− 1.51	− 1.15	− 1.04	Destabilizing
4	V666L	− 0.69	− 0.09	− 0.41	− 0.54	− 0.15	Destabilizing

^**a**^Web server that integrates two computational approaches (mCSM and SDM) to generate a consensus protein stability prediction

^**b**^Prediction from different template structures: 4ZGC is an S–S linked structure, and 4YY8 is an unlinked structure

ΔΔG, changes in stability due to mutations; mCSM, mutation cutoff scanning matrix; SDM, site‐directed mutator; PDB, Protein Data Bank

### Pfhrp2 and pfhrp3 deletions

*Pfhrp2/pfhrp3* deletion analysis was performed in 36 RDT-negative but PCR-positive samples. Among these, 2/36 (5.6%) were positive for *pfhrp2*, and 9/36 (25.0%) had complete *pfhrp2* deletions. There were 13/36 (36.1%) samples with partial deletions in both the upstream and downstream regions of *pfhrp2*. In addition, 7/36 (19.4%) were in the upstream region only, while 5/36 (13.9%) showed deletions involving both the upstream region and exon 2. There were 11/36 (30.6%) samples positive for *pfhrp3*, followed by 10/36 (27.8%) with complete *pfhrp3* deletions. Various partial gene deletion patterns were present in both *pfhrp2* and *pfhrp3* (Table 3). Notably, among samples with parasite densities greater than 200 parasites/µL, both RDT-positive and RDT-negative results were observed (Table 4). When combining *pfhrp2*/*pfhrp3* deletion patterns (Table 5), the most common pattern was partial deletions of both genes (14/36; 38.9%), followed by dual deletions (7/36; 19.4%). In contrast, dual-positive genes were only detected in 2/36 (5.6%) samples.

**Table 3:** Combination patterns of *pfhrp2*, *pfhrp3*, and flanking gene deletions in RDT-negative, *P. falciparum*-positive samples collected in Tanzania in 2023

Pattern	*pfhrp2 gene*			Multiplex PCR/Edwin Pf		N (%)	*Pfhrp3 gene*			Multiplex PCR/Edwin Pf		N (%)
	MALP1.230 (upstream)	*pfhrp2 Exon2*	MALP1.228 (upstream)	*Exon2*	Edwin		MAL13PI.475 (upstream)	*Pfhrp3 Exon2*	MAL13PI.485 (downstream)	*Exon2*	Edwin	
1	+	+	+	−	−	2(5.6)	+	+	+	+	+	1(2.8)
2	−	+	+	+	+	1(2.8)	+	+	+	−	+	2(5.6)
3	−	+	+	−	+	1(2.8)	+	+	+	−	−	8(22.2)
4	−	+	+	−	−	5(13.9)	+	+	−	−	+	1(2.8)
5	−	+	−	−	+	2(5.6)	+	+	−	−	−	1(2.8)
6	−	+	−	−	−	11(30.6)	+	−	−	−	−	3(8.3)
7	−	−	+	−	−	5(13.9)	−	+	+	−	−	1(2.8)
8	−	−	−	−	−	9(25)	−	+	−	−	−	8(22.2)
9							−	−	+	−	−	1(2.8)
10							−	−	−	−	−	10(27.8)
Total						36(100)						36(100)

**Table 4:** Relationship between HRP2-RDT results and parasite density determined by qPCR

HRP2-RDT	qPCR		
	Neg	0–200 parasites/μL	> 200 parasites/μL
Negative RDT	n = 0	n = 34	n = 2
Positive RDT	n = 0	n = 28	n = 2

**Table 5:** Incidence of *pfhrp2/pfhrp3* deletion patterns in RDT-negative, *P. falciparum*-positive samples collected in Tanzania in 2023

Gene		Number of samples (N)	Proportions (%)
*Pfhrp2*	*Pfhrp3*		
+	+	2	5.6
Partial *hrp2* gene deletion	+	9	25
Partial *hrp2* gene deletion	Partial *hrp3* gene deletion	14	38.9
Partial *hrp2* gene deletion	−	4	11.1
−	−	7	19.4

### 18S rRNA multiplex PCR and *pfhrp2/3* exon 2 verification and comparison with RDT results

**Fig. 4:**
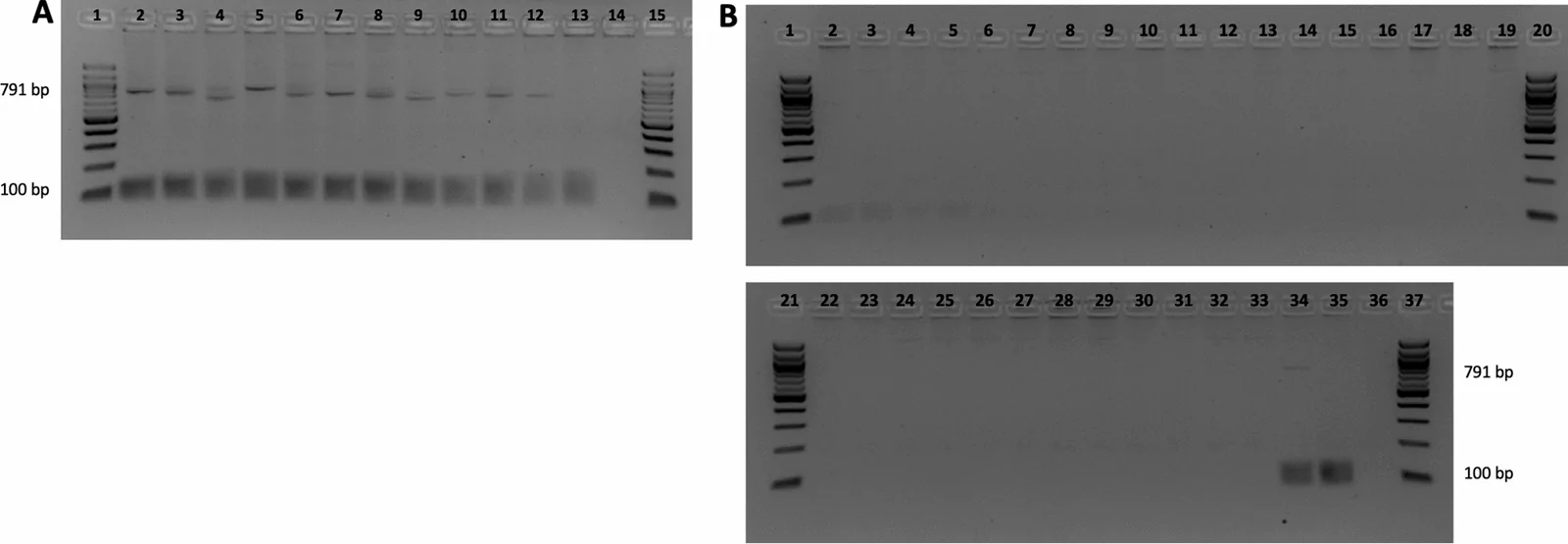
**A** Multiplex PCR amplification of multiplex *hrp2* PCR from RDT-positive *P. falciparum* isolates with parasitemia ranging from 16,700 to 267,562 parasites/μL. Lanes 1 and 15: 100-bp DNA ladder, lanes 2–11: samples; lane 12: positive control (3D7), lane 13: Dd2 (*hrp2*-negative laboratory strain), and lane 14: negative PCR control. The expected size of the amplified exon 2 product is 791 bp; lower bands correspond to *P. falciparum* parasite products. **B** Amplification of multiplex *hrp2* PCR from RDT-positive samples with low levels of parasitemia (0.17–518 parasites/μL). Lanes 1, 20, 21 and 37: 100-bp DNA ladder, Lanes 2–19 and 22–33: samples; lane 34: positive control (3D7), lane 35: Dd2 (*hrp2*-negative laboratory strain), and lane 36: negative PCR control

All 36 isolates were re-analyzed using multiplex PCR targeting 18S rRNA and *pfhrp2* exon 2 region. Exon 2 deletions were assessed by comparing PCR results across distinct sample groups. The first group consisted of *P. falciparum* isolates with parasite densities ranging from 16,700 to 267,562 parasites/µL. The second group included samples with high parasite densities (≥ 10,000 parasites/µL). The third group comprised 30 RDT-positive samples with lower parasite densities (0.17–518 parasites/µL), selected for comparative analysis. In the high parasitemia group (≥ 10,000 parasites/µL), all samples were positive for both 18S rRNA and *pfhrp2/3* exon 2 targets, confirming successful parasite detection (Fig. 4a). In contrast, among the 30 RDT-positive low-parasitemia samples, only one sample was positive for *pfhrp2* exon 2, and four samples were positive for 18S rRNA, while the remaining samples were negative for both targets (Fig. 4b).

**Fig. 5:**
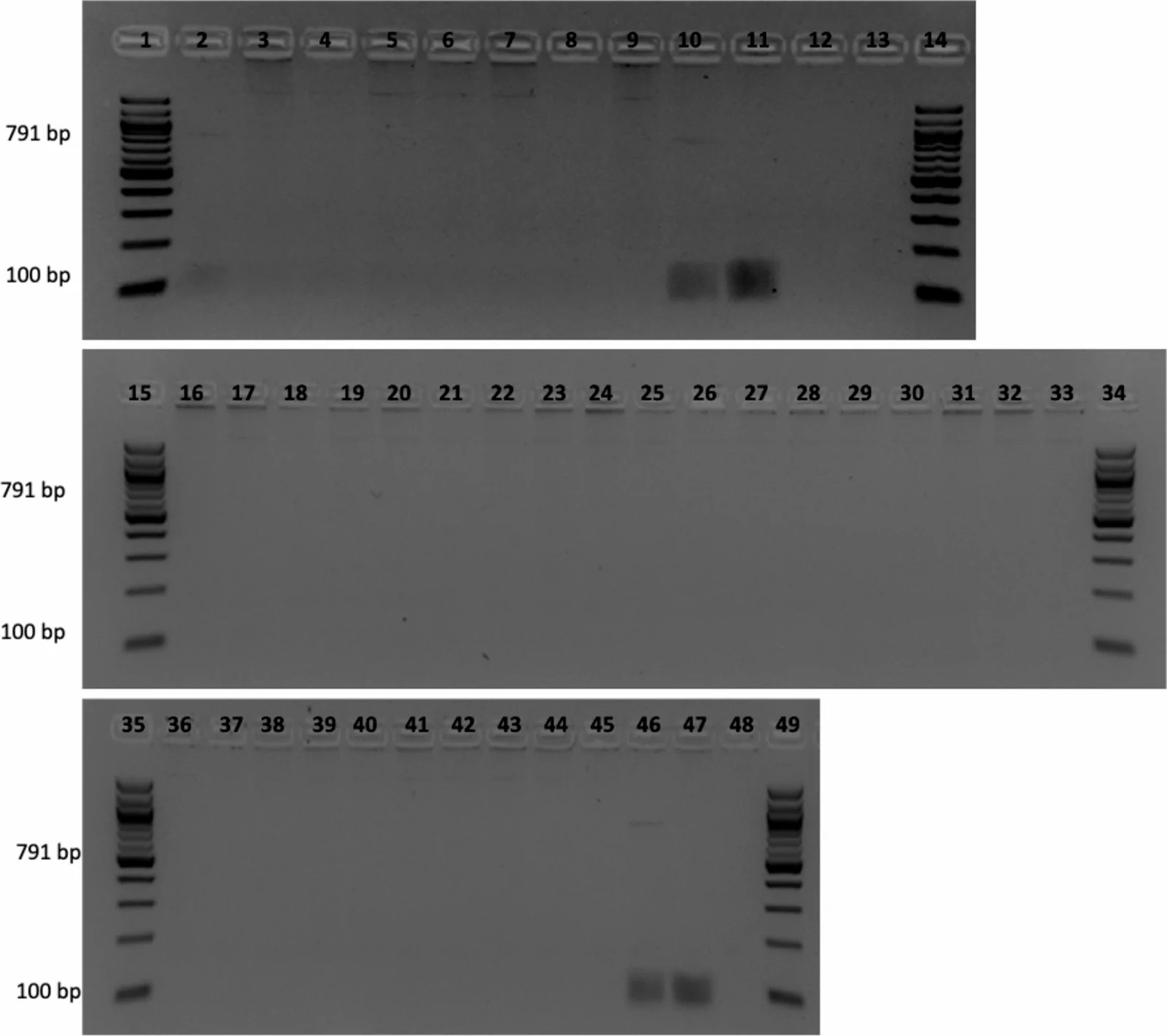
Multiplex PCR amplification of *hrp*2 from RDT-negative samples confirmed as infected with *P. falciparum*, with parasitemia ranging from 0.26 to 684 parasites/μL. Lane 1, 14–15, 34–35 and 49: 100-bp DNA ladder, lanes 2–9, 16–33 and 36–45: samples, lane 10 and 46: positive control (3D7), lane 11 and 47: Dd2 (*hrp2*-negative laboratory strain), lane 12–13 and 48: negative PCR control. The expected size of the amplified exon 2 product is 791 bp and *P. falciparum* product is 100 bp

Similarly, among 36 RDT-negative samples confirmed as infected with *P. falciparum*, one was positive for *pfhrp2* exon 2 and four were positive for 18S rRNA PCR (Fig. 5), with parasitemia ranging from 0.26 to 684 parasites/μL. However, some samples with parasitemia below the RDT detection limit were negative despite retaining *pfhrp2*, indicating false negatives resulting from low antigen concentrations rather than gene deletions. Conversely, samples with parasitemia above the detection limit but negative for HRP2 antigen (i.e., RDT-negative) are more likely to represent true *pfhrp2* deletions or markedly reduced expression.

In accordance with WHO recommendations for the confirmation of *pfhrp2/3* gene deletions using a previously described method by Grignard et al. [30], the results from both assays were fully concordant. For *pfhrp2* exon 2, 18 samples (50.0%) were classified as positive, while for *pfhrp3* exon 2, 20 samples (55.6%) were positive.

## Discussion

Early diagnosis and treatment, combined with chemopreventive strategies, such as IPTp, IPTi, and SMC, are crucial for malaria control [33–35]. In Tanzania, the first-line treatment for uncomplicated *P. falciparum* is artemether/lumefantrine, while SP is still used for IPTp, IPTi, and SMC, maintaining selective pressure on parasite populations [36–38].

During the study period (2021–2024), changes in the distribution of pfdhfr triple mutant and mixed haplotypes were observed; however, the overall prevalence of C59R remained relatively stable. These findings suggest temporal changes in haplotype composition rather than clear directional selection under antifolate drug pressure. Compared with earlier reports from Mtwara [38, 39], which showed high prevalence of triple mutants (74% in 2006–2007 and 92% in 2010–2011), the current results indicate changes in genotype distribution over time, although triple mutants remain common in the study area. These patterns may also be influenced by differences in transmission intensity and multiplicity of infection (MOI), which were not directly assessed in this study. Previous studies have reported the distribution of *pfdhfr* haplotypes in Tanzania and other parts of Africa; however, the available evidence is insufficient to determine the exact origin and global dissemination pathways of mutations such as S108N [40].

The resistance-associated *pfdhps* double mutation A437G and K540E was highly prevalent, increasing from 59.4 to 70.8% over the study period, consistent with sub-Saharan African trends and SP treatment failure [41, 42]. When *pfdhfr* and *pfdhps* mutations were combined, quintuple mutants (AIRNI + ISGEAA) were observed at high frequencies in 2021–2022 (35.1% and 37.5%, respectively), declining in 2023–2024 (23.9% and 20.8%, respectively). In contrast, mixed haplotypes (AIC/RNI + ISGEAA) increased from 28.3% to 40.6% between 2023 and 2024, suggesting shifts in parasite population structure. The persistence of resistant alleles probably reflects multiple factors, including SMC, IPTp use, self-medication, and cross-resistance with other antifolate drugs [36, 43]. Although the declining rates of the *pfdhfr* triple mutant haplotype (N51I/C59R/S108N) and the *pfdhfr/pfdhps* quintuple mutant haplotype (N51I, C59R, and S108N + A437G and K540E) are encouraging, resistant alleles persist, particularly in the context of SMC and IPTp. The integration of molecular marker-based monitoring with efficacy assessments of preventive and therapeutic interventions is crucial to inform national malaria treatment policies. Furthermore, the detection of potential increases in resistant haplotypes will serve as a warning of impending treatment failures, highlighting the complex evolutionary dynamics of antifolate resistance.

The emergence of artemisinin resistance was first reported in Southeast Asia and more recently in Rwanda. Artemisinin resistance is confirmed by characteristic in vivo or in vitro phenotypes and shows a strong association with mutations in the propeller domain of *pfkelch13* [7, 44, 45]. Well-characterized mutations, including Y493H, R539T, I543T, and C580Y, are prevalent in the Greater Mekong Subregion but remain uncommon in Africa [27, 46, 47]. *Pfkelch13* polymorphisms are often detected in Africa; Southeast Asian *P. falciparum* resistance-associated alleles have not been widely observed [12, 48, 49]. R561H has been detected in Rwanda and Tanzania, and A675V and C469Y in Uganda [9, 50, 51]. Common African variants, such as K189T and K189N, which are located outside the propeller domain, are not associated with delayed parasite clearance [52]. None of the Tanzanian isolates in our study harbored validated *pfkelch13* resistance mutations. However, previous studies have reported the presence of mutations such as R561H in Tanzania and neighboring East African countries [53], indicating regional variation in K13 mutation frequencies. However, we observed nine haplotypes with synonymous mutations, seven with nonsynonymous mutations, and several mixed wild-type variants. The synonymous allele C469C was observed at frequencies similar to those in other African countries [54–57]. Nevertheless, the detection of R561H (which confers partial resistance to artemisinin) in Rwanda and southeastern Tanzania [50, 58] underscores the importance of continued surveillance.

Our findings have implications for malaria diagnosis using HRP2-based RDT testing. Such tests are used in Tanzania because they are easy to use and highly sensitive at moderate to high levels of parasitemia [59, 60]. However, deletions in *pfhrp2* or *pfhrp3* reduce HRP2 antigen production, which may lead to false-negative results. Furthermore, parasite density strongly influences diagnostic performance; *HRP2*-based RDTs typically detect ≥ 50 parasites/µL, whereas real-time PCR detects as few as 0.1–1 parasites/µL [15]. Among the 36 RDT-negative, PCR-confirmed *P. falciparum* samples analyzed, partial *pfhrp2* deletions were detected in 69.4% and *pfhrp3* deletions in 41.7%. Partial deletions affecting both genes were the most common pattern (38.9%), followed by dual complete *pfhrp2/3* deletions (19.4%), while intact dual-positive genes were detected in 5.6% of samples. These findings describe the distribution of pfhrp2 and pfhrp3 deletion patterns within the selected study samples. Our results also showed a notable proportion of partial deletions, particularly in the upstream regions of *pfhrp2*. Such partial deletions may disrupt gene integrity or antigen production, which may have implications for HRP2 detection by RDTs and could contribute to variability in test performance. This underscores the importance of using molecular surveillance to distinguish between complete and partial deletions [18, 19]. In our study, the incidence of *pfhrp3* deletions and overlapping *pfhrp2/pfhrp3* deletions further complicates diagnostic reliability. *pfhrp3* shares epitopes with *HRP2*, and its loss may eliminate compensatory antigen production, which helps to sustain RDT sensitivity in strains with *pfhrp2* deletion [16]. Our data suggest that simultaneous deletion of *pfhrp2* and *pfhrp3* significantly undermines RDT performance. These findings align with reports from East Africa, which also document increasing frequencies of *pfhrp2* deletion in areas with low-to-moderate transmission rates [17, 61, 62]. Collectively, the high rates of complete and partial deletions suggest the need for alternatives to *hrp2/3*-based tests as frontline diagnostic tools in Tanzania.

A potential limitation of our study is the use of multiplex PCR to detect *pfhrp2/3* deletions because samples with low levels of parasitemia may not test positive due to undetectable gene signals. However, nested PCR is more sensitive and has a lower limit of detection, making it a more reliable method for confirming *pfhrp2* deletions.

## Conclusions

The decline in *pfdhfr* triple mutations alongside the rise in mixed wild-type haplotypes of *P. falciparum* in Tanzania between 2021 and 2024 suggests shifts in parasite population structure. The persistence of *pfdhps* double and quintuple mutations continues to confer and maintain SP resistance, which may impact chemopreventive interventions, such as IPTp and SMC. No *pfkelch13* mutations associated with artemisinin resistance were detected. The incidence of *pfhrp2/3* false-negative results and deletions highlights the potential limitations of *HRP2*-based RDTs. Collectively, these findings emphasize the need for ongoing molecular surveillance to monitor resistant alleles, mixed infections, and gene deletions. Such data are crucial for guiding malaria treatment policies and public health strategies in Tanzania.

## Supplementary Information

Additional file 1.

Additional file 2.

## Data Availability

Data supporting the findings of this study are available from the corresponding author on reasonable request.

## Abbreviations

DBS: Dried blood spot
IPT: Intermittent preventive therapy
IPTi: Intermittent preventive therapy in infancy
IPTp: Intermittent preventive therapy in pregnancy
PCR: Polymerase chain reaction
RDT: Rapid diagnostic test
SMC: Seasonal malaria chemoprophylaxis
SP: Sulfadoxine-pyrimethamine
