# Supplementary material for: Molecular characterization of antimalarial resistance and hrp2/3 deletions in Plasmodium falciparum strains from Mtwara, Tanzania (2021–2024)
Source: Malar J. 2026 Jul 28;25:377. doi: 10.1186/s12936-026-06028-2 (PMC13700497; doi:10.1186/s12936-026-06028-2)
Supplement: Supplementary file 1 [file 12936_2026_6028_MOESM1_ESM.pdf]

Molecular characterization of *Plasmodium falciparum* in Tanzania

Supplementary material

Table S1 Primer sequences for detection *Plasmodium* species, SP and artemisinin resistance markers in *P. falciparum*.

Detections	Primers/Probes	Sequence (5'-3')	Product size (bp)
**Nest1-*pfdhfr***	*pfdhfr*_PrF	ATGGARSAMSTYTSMGABGTWTTY [ATGGA(G/A)(G/C)A(A/C)(G/C)T(T/C)T(G/C)(A/C)GA(G/T/C)GT(A/T)TTY]	714
	*pfdhfr*_M5	AGTATATACATCGCTAACAGA	
**Nest2-*pfdhfr***	*pfdhfr*_PrNF	TSMGABGTWTTYGAYATTTAYGC [T(G/C)(A/C)GA(G/T/C)GT(A/T)TT(T/C)GA(T/C)ATTTA(T/C)GC]	710
	*pfdhfr*_M5	AGTATATACATCGCTAACAGA	
**Nest1-*pfdhps***	*pfdhps*_F	GTTGAACCTAAACGTGCTGT	880
	*pfdhps*_R/	AATTGTGTGATTTGTCCACAA	
**Nest2-*pfdhps***	*pfdhps*_R2	AACCTAAACGTGCTGTTCAA	648
	*pfdhps*_R/	AATTGTGTGATTTGTCCACAA	
**Nest1-*pfkelch13***	K13_c.1F	TGGAAGGAGAAAAAGTAAAAACAAAA	2283
	K13_c.2283R	TGTGCATGAAAATAAATATTAAAGAAG	
**Nest2-*pfkelch13* (F1&F2)**	K13_K189T_F	ACCAAGTAATTTATCAAGAACATTT	1245
	K13_c.1538R	CGATCATACACCTCAGTTTCAA	
**Nest2-*pfkelch13* (F3)**	K13_c.1344F	AGGTGGATTTGATGGTGTAGAA	940
	K13_c.2283R	TGTGCATGAAAATAAATATTAAAGAAG	
**Multiplex hrp2 exon 2 / Pf**	Pfhrp2exon2N2F	ATTATTACACGAAACTCAAGCAC	791
	Pfhrp2exon2N1R	AATAAATTTAATGGCGTAGGCA	
	EdwinFor	GCTCTTTCTTGATTTCTTGGATG	100
	EdwinRe	AGCAGGTTAAGATCTCGTTCG	

Table S2 The prevalence reports of *pfdhfr* mutations in study site, Tanzania between 2021 and 2024.

Years	*Pfdhfr* gene					Count (N)	Proportions (%)
	A16V	N51I	C59R	S108N	I164L		
2021	A	N	C	A	I	1	1.1
(n = 99)	A	N	R	N	I	5	5.1
	A	I	C	N	I	5	5.1
	A	I	R	N	I	61	61.6
	A	I	C/R	N	I	18	18.2
	A	N/I	R	N	I	6	6.1
	A	N/I	C/I	N	I	3	3
2022	A	N	R	N	I	11	10.2
(n = 108)	A	I	C	N	I	10	9.3
	A	I	R	N	I	56	51.9
	A	N	C/R	N	I	4	3.7
	A	I	C/R	N	I	23	21.3
	A	N/I	C/R	N	I	2	1.9
	A	I	C/R	S/N	I	2	1.9
2023	A	N	R	N	I	4	4
(n = 99)	A	I	C	N	I	6	6.1
	A	I	R	N	I	37	37.4
	A	N	C/R	N	I	2	2
	A	I	C/R	N	I	42	42.4
	A	N/I	R	N	I	5	5.1
	A	N/I	C/R	N	I	3	3
2024	A	N	C	S	I	2	1.9
(n = 108)	A	N	R	N	I	3	2.8
	A	I	C	N	I	3	2.8
	A	I	R	N	I	33	30.6
	A	N	C/R	N	I	4	3.7
	A	I	C/R	N	I	59	54.6
	A	N/I	R	N	I	2	1.9
	A	N/I	C/R	N	I	1	0.9
	A	I	C/R	S/N	I	1	0.9

Table S3 The prevalence reports of *pfdhps* mutations in study site, Tanzania between 2021 and 2024.

Years	*Pfdhps* gene						Count (N)	Proportions (%)
	I431V	S436F/A	A437G	K540E	A581G	A613S/T		
2021	I	S	A	K	A	A	8	8.3
(n = 96)	I	S	A	E	A	A	1	1
	I	A	A	K	A	A	2	2.1
	I	H	A	K	A	A	2	2.1
	I	F	A	K	A	S	1	1
	I	F	A	E	A	S	1	1
	I	S	G	E	A	A	57	59.4
	I	S	A	K/E	A	A	1	1
	I	C	A	K/E	A	A	1	1
	I	S	G	K/E	A	A	2	2.1
	I	S	A/G	E	A	A	5	5.2
	I	S	A/G	K/E	A	A	11	11.5
	I	S/A	A/G	K/E	A	A	1	1
	I	S/G	A/G	K/E	A	A	1	1
	I	S/F	A/G	K/E	A	A	1	1
	I	S/F	A/G	K/E	A	A/S	1	1
2022	I	S	A	K	A	A	13	11.8
(n = 110)	I	A	A	K	A	A	2	1.8
	I	H	A	K	A	A	2	1.8
	I	F	A	K	A	A	2	1.8
	I	A	G	K	A	A	1	0.9
	I	S	G	E	A	A	74	67.3
	I	S	G	E	G	A	1	0.9
	I	S	A	K/E	A	A	1	0.9
	I	S	A/G	K	A	A	1	0.9
	I	S	G	K/E	A	A	1	0.9
	I	S	A/G	E	A	A	4	3.6
	I	S	A/G	K/E	A	A	5	4.6
	I	S/A	A/G	K/E	A	A	1	0.9
	I	S/Y	A/G	K/E	A	A	1	0.9
	I	S/F	A/G	K/E	A	A/S	1	0.9

Table S3 The prevalence reports of *pfdhps* mutations in study site, Tanazia between 2021 and 2024 (cont.).

Years	*Pfdhps* gene						Count (n)	Proportions (%)
	I431V	S436F/A	A437G	K540E	A581G	A613S/T		
2023	I	S	A	K	A	A	4	4.3
(n = 93)	I	A	A	K	A	A	2	2.2
	I	H	A	K	A	A	1	1.1
	I	P	A	K	A	A	1	1.1
	I	S	G	E	A	A	63	67.7
	I	F	A	E	A	S	1	1.1
	I	S	A	K/E	A	A	2	2.2
	I	S	A/G	E	A	A	2	2.2
	I	S	A/G	K/E	A	A	14	15.1
	I	A	A/G	K/E	A	A	1	1.1
	I	S/C	A/G	K/E	A	A	1	1.1
	I	S/A	A/G	K/E	A	A	1	1.1
2024	I	S	A	K	A	A	9	8.5
(n = 106)	I	A	A	K	A	A	3	2.8
	I	C	A	K	A	A	1	0.9
	I	H	A	K	A	A	7	6.6
	I	S	A	E	A	A	1	0.9
	I	F	A	K	A	S	1	0.9
	I	S	G	E	A	A	75	70.8
	I	S	A/G	K	A	A	1	0.9
	I	S	A/G	E	A	A	1	0.9
	I	S	G	K/E	A	A	2	1.9
	I	S	A/G	K/E	A	A	5	4.7

Table S4 The prevalence of *pfdhfr/dhps* combination haplotypes in Tanzania between 2021 and 2024.

Category	Haplotype	% Frequency			
		2021 (n = 94)	2022 (n = 104)	2023 (n = 92)	2024 (n = 106)
Wild type	ANCSI + ISAKAA	1.1			
Single mutant	ANCSI + IHAKAA				0.9
Double mutant	ANCSI + ISGEAA				0.9
	ANRNI + ISAKAA		1		
	AICNI + ISAKAA		1	1.1	
Triple mutant	ANRNI + IFAKAA		1		
	ANRNI + IHAKAA		1		0.9
	ANRNI + ISAK/EAA		1	1.1	
	ANC/RNI + ICAKAA				0.9
	AN/IRNI + ISAKAA				0.9
	AICNI + IAAKAA		1		
	AICNI + IHAKAA	3.2			
	AICNI + ISAK/EAA	1.1			
	AIRNI + ISAKAA	3.2	7.7	2.2	5.7
	AIC/RNI + ISAKAA	1.1	2.9	1.1	1.9
Quadruple mutant	ANRNI + ISGEAA	4.3	4.8	3.3	1.9
	ANRNI + ISA/GK/EAA		1.9		
	ANC/RNI + IAGKAA		1		
	ANC/RNI + ISGEAA		1.9	2.2	1.9
	ANC/RNI + ISA/GK/EAA		1		
	ANC/RNI + ISGK/EAA				0.9
	AN/IRNI + IAAKAA				0.9
	AN/IRNI + IPAKAA			1.1	
	AICNI + ISGEAA	4.3	6.7	4.4	2.8
	AIRNI + IAAKAA	2.1	1	2.2	
	AIRNI + IFAKAA		1		
	AIRNI + IHAKAA	2.1	1	1.1	0.9
	AIRNI + ISAEAA	1.1			0.9

Table S4 The prevalence of *pfdhfr/dhps* combination haplotypes in Tanzania between 2021 and 2024 (cont).

Category	Haplotype	% Frequency			
		2021 (n = 94)	2022 (n = 104)	2023 (n = 92)	2021 (n = 94)
	AIRNI + ISA/GEAA		1.9		
	AIRNI + ISA/GK/EAA		1		
	AIRNI + ISA/GKAA		1		
	AIC/RNI + IAAKAA				1.9
	AIC/RNI + IHAKAA				3.8
	AIC/RNI + ISA/GKAA				0.9
	AIC/RNI + ISAK/EAA			1.1	
Quintuple mutant	AICNI + IFAEAS	1.1		1.1	
	AICNI + IS/AA/GK/EAA		1		
	AIRNI + IFAKAS	1.1			0.9
	AIRNI + ISGEAA	1.1	37.5	23.9	20.8
	AIRNI + ICAK/EAA	35.1			
	AIRNI + ISA/GEAA	1.1		1.1	0.9
	AIRNI + ISGK/EAA	3.2	1		0.9
	AIRNI + ISA/GK/EAA	1.1		5.4	0.9
	AN/IRNI + ISA/GEAA	9.6		1.1	
	AN/IRNI + ISGEAA	2.1		3.3	
	AN/IC/RNI + ISA/GK/EAA	4.3			
	AN/IC/RNI + ISGEAA	1.1		2.2	0.9
	AN/IC/RNI + ISA/GEAA		0.96		
	AIC/RNI + ISGEAA	1.1	12.5	28.3	40.6
	AIC/RNI + ISGK/EAA	11.7			
	AIC/RNI + ISA/GEAA		1		
	AIC/RNI + ISA/GK/EAA	1.1	1	9.8	3.8
	AIC/RS/NI + ISGEAA		1.9		0.9
Sextuple mutant	AIRNI + IS/FA/GK/EAA	1.1			
	AN/IC/RNI + IS/GA/GK/EAA	1.1			
	AIC/RNI + IS/AA/GK/EAA	1.1			

Table S4 The prevalence of *pfdhfr/dhps* combination haplotypes in Tanzania between 2021 and 2024 (cont).

Category	Haplotype	% Frequency			
		2021 (n = 94)	2022 (n = 104)	2023 (n = 92)	2024 (n = 106)
Septuple mutant	AIC/RNI + IAA/GK/EAA			1.1	
	AIC/RNI + IS/CA/GK/EAA			1.1	
	AIC/RNI + ISGEGA		1	1.1	
	AIC/RNI + IS/YA/GK/EAA		1		
	AIRNI + IS/FA/GK/EAA/S	1.1			
	AIC/RNI + IS/FA/GK/EAA/S		1		

Table S5 The prevalence reports of *pfkelch13* mutations in study site, Tanzania between 2021 and 2024.

Gene	Year	Haplotype	Count (N)	Percentage (%)
Pfkelch13	2021	Wild type	22	75.9
	n = 29	**NN** at codon 142	1	3.4
		K189**T**	6	20.7
	2022	Wild type	38	63.3
	n = 60	**N** at codon 142	1	1.7
		**NN** at codon 142	3	5
		**NNN** at codon 142	1	1.7
		I132**S**	1	1.7
		K189**N**	1	1.7
		K189**T**	9	15
		K189**T/K**	1	1.7
		T192**P/T**	1	1.7
		R255**K**	1	1.7
		R398**R**	1	1.7
		P417**P**	1	1.7
		C469**C**	1	1.7
	2023	Wild type	69	65.1
	n = 106	**N** at codon 142	5	4.7
		**NN** at codon 142	2	1.9
		H136**N**	1	0.9
		K189**T**	8	7.6
		G220**G**	1	0.9
		K189**T/K**	13	12.3
		R223**K/R**	1	0.9
		N138**N/N**, K189**T**	1	0.9
		K189**T/K**, L251**L**	1	0.9
		G220**S/G**, G220**F/G**	1	0.9
		C469**C**	1	0.9
		V666**L/V**	1	0.9
		K189**N/K**, C469**C/C**	1	0.9

Table S5 The prevalence reports of *pfkelch13* mutations in study site, Tanzania between 2021 and 2024 (cont.).

Gene	Year	Haplotype	Count (N)	Percentage (%)
	2024	Wild type	135	61.6
	n = 219	**N** at codon 142	4	1.8
		**NN** at codon 142	4	1.8
		**NNN** at codon 142	1	0.5
		K189**N**	1	0.5
		K189**N/K**	2	0.9
		K189**T**	29	13.2
		K189**T**, L222**L**	1	0.5
		K189**T/K**	16	7.3
		L258**L/M**	1	0.5
		L152**L/L**	1	0.5
		M157**V**	1	0.5
		L258**M**	2	0.9
		Q271**Q**	1	0.5
		R255**K**	3	1.4
		R398**R**	1	0.5
		P417**P**	1	0.5
		C469**C**	1	0.5
		A504**A**	1	0.5
		G538**G**	1	0.5
		P715**P**	1	0.5
		C469**C/C**	2	0.9
		C473**P/C**	1	0.5
		P574**S/P**	1	0.5
		A578**S/A**	1	0.5
		A676**A/A**	1	0.5
		K189**N**, C469**C**	2	0.9
		K189**T**, C469**C**	1	0.5
		K189**T**, P441**P/P**, P443**P/P**	1	0.5
		K189**T**, N594**S/N**, V647**I**	1	0.5

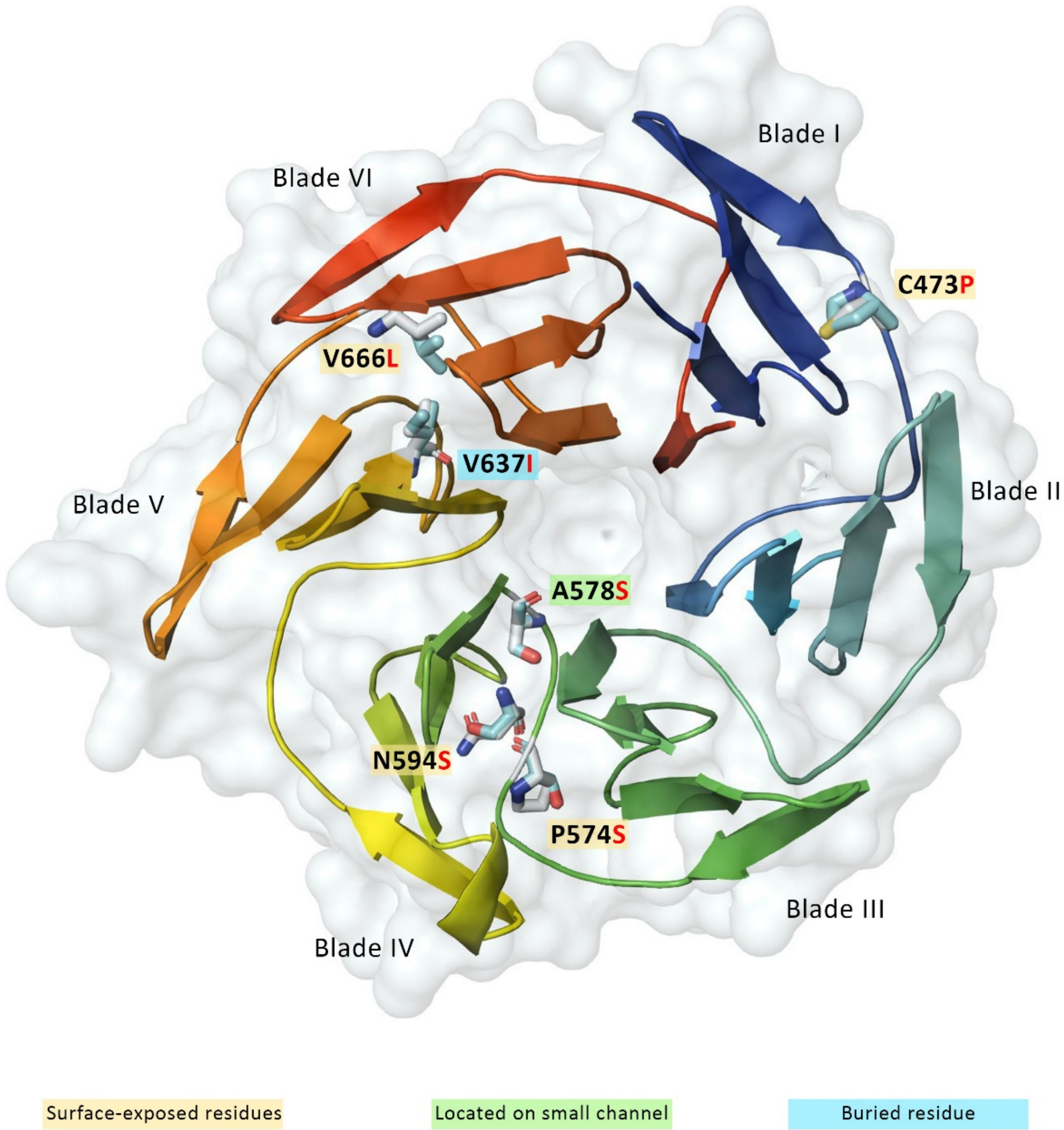

Figure S1. The built K13 propeller domain (amino acid 442nd – 726th) structure of *P. falciparum* from Tanzania. The six detected mutations were shown as a cyan stick, while the wild type was represented as a grey stick. The location of mutated residues on the structure was highlighted in yellow, green, and blue boxes for surface-exposed, close to the small channel, and buried residues, respectively.
