# Supplementary material for: Molecular characterization of antimalarial resistance and hrp2/3 deletions in Plasmodium falciparum strains from Mtwara, Tanzania (2021–2024)
Source: Malar J. 2026 Jul 28;25:377. doi: 10.1186/s12936-026-06028-2 (PMC13700497; doi:10.1186/s12936-026-06028-2)
Supplement: Supplementary file 2 [file 12936_2026_6028_MOESM2_ESM.docx]

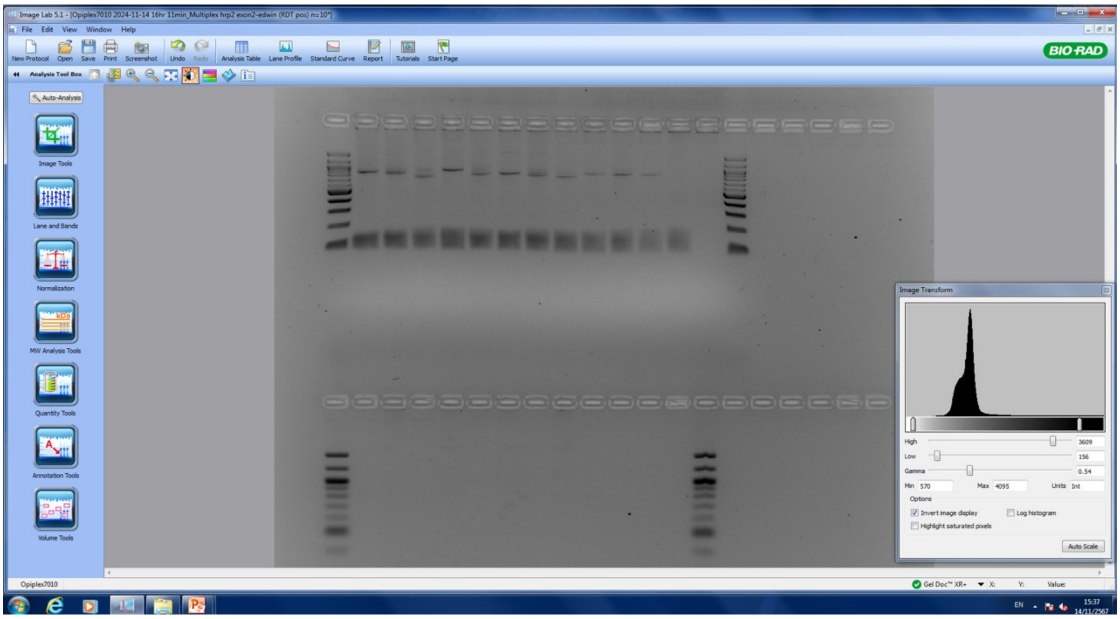
Image Lab 5.1 - [Opiplex7010 2024-11-14 16hr 11min_Multiplex hrp2 exon2-edwin (RDT pos) n=10*]
BIO-RAD
Image Transform
Invert image display
Log histogram
Highlight saturated pixels
Auto Scale
Opiplex7010

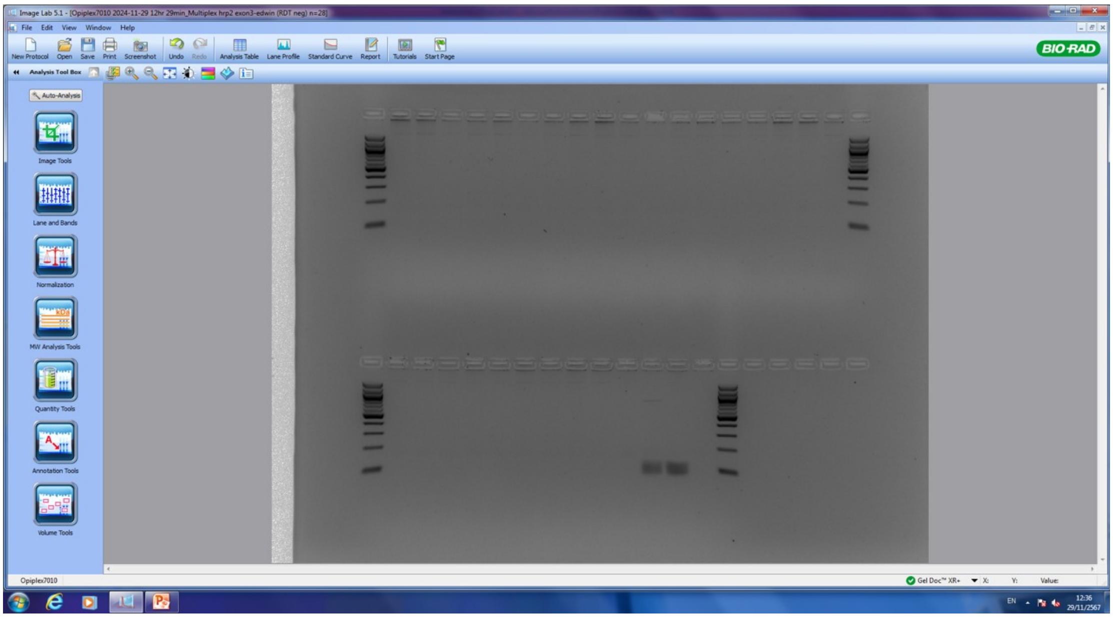
Image Lab 5.1 - [Opiplex7010 2024-11-29 12hr 29min_Multiplex hrp2 exon3-edwin (RDT neg) n=28]
BIO-RAD
Opiplex7010

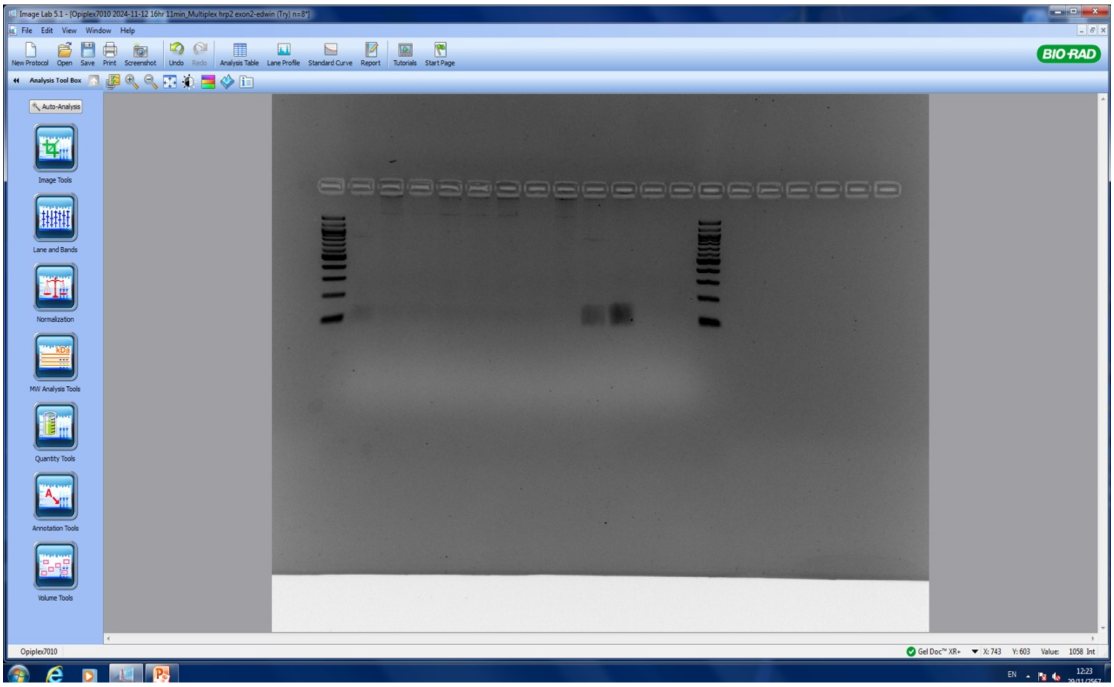
Image Lab 5.1 - [Opiplex7010 2024-11-12 16hr 11min_Multiplex hrp2 exon2-edwin (Try) n=8*]
File Edit View Window Help
New Protocol Open Save Print Screenshot Undo Redo Analysis Table Lane Profile Standard Curve Report Tutorials Start Page
BIO-RAD
Analysis Tool Box
Auto-Analysis
Image Tools
Lane and Bands
Normalization
MW Analysis Tools
Quantity Tools
Annotation Tools
Volume Tools
Opiplex7010
Gel Doc™ XR+
X: 743 Y: 603 Value: 1058 Int
EN
12:23

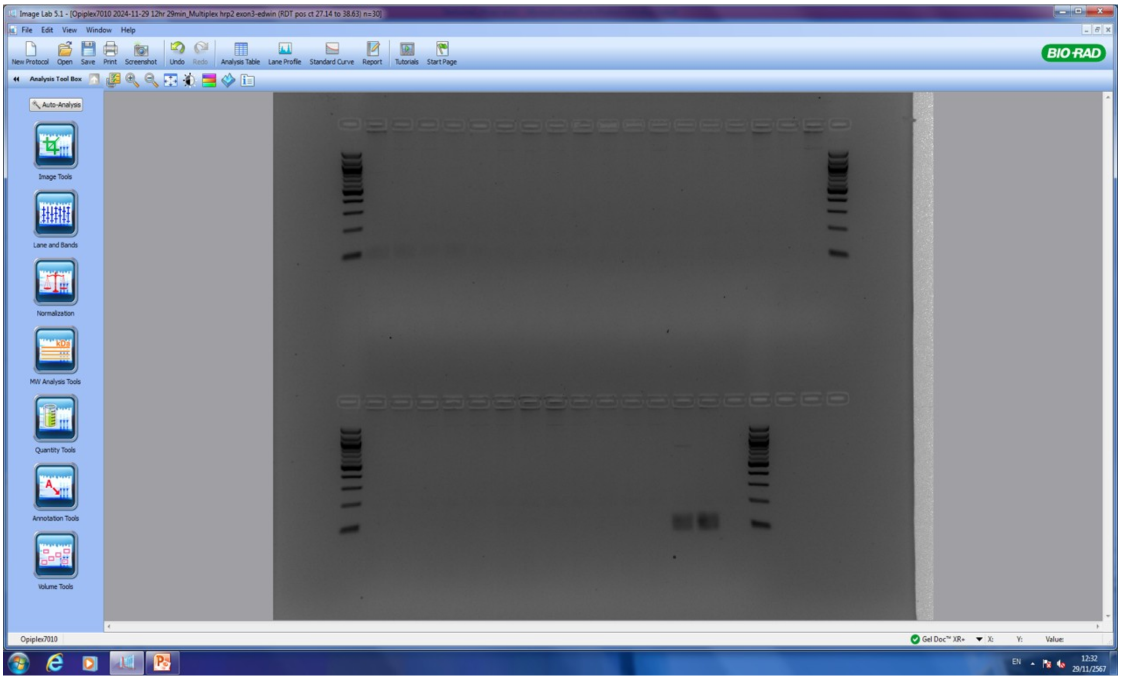
Image Lab 5.1 - [Opiplex7010 2024-11-29 12hr 29min_Multiplex hrp2 exon3-edwin (RDT pos ct 27.14 to 38.63) n=30]
File Edit View Window Help
New Protocol Open Save Print Screenshot Undo Redo Analysis Table Lane Profile Standard Curve Report Tutorials Start Page
BIO-RAD
Analysis Tool Box
Auto-Analysis
Image Tools
Lane and Bands
Normalization
MW Analysis Tools
Quantity Tools
Annotation Tools
Volume Tools
Opiplex7010
Gel Doc™ XR+
X: Y: Value:
EN
12:32
29/11/2567
